# FRY, a global database of fire patch functional traits derived from space-borne burned area products

**DOI:** 10.1038/sdata.2018.132

**Published:** 2018-07-10

**Authors:** Pierre Laurent, Florent Mouillot, Chao Yue, Philippe Ciais, M. Vanesa Moreno, Joana M. P. Nogueira

**Affiliations:** 1 Laboratoire des Sciences du Climat et de l'Environnement, CEA-CNRS-UVSQ, UMR8212 Gif-sur-Yvette, France; 2 UMR CEFE 5175, Centre National de la Recherche Scientifique (CNRS), Université de Montpellier, Université Paul-Valéry Montpellier, Ecole Pratique des Hautes Etudes (EPHE), Institut de Recherche pour le Développement (IRD), 1919 route de Mende, 34293 Montpellier CEDEX 5, France; 3 National Institute for Space Research (INPE) Department of Meteorology, room 14 Av. dos Astronautas, 1758, Jardim da Granja São José dos Campos State of São Paulo, 12227-010 SP, Brazil

**Keywords:** Fire ecology, Carbon cycle

## Abstract

Vegetation fires are intrinsic ecosystem disturbances of the Earth system. Global burned area products have been delivered from several space-borne instruments, and have recently provided pixel-level information underpinning fire spread processes. Here we present FRY, a global database of fire patches with morphology-based functional traits reconstructed from pixel-based burned areas derived from the MODIS and MERIS imagery using a flood-fill algorithm. Each fire patch is characterized by the geo-location of its center, area, perimeter, the features of the ellipse fitted over its pixel’s spatial distribution, and different indices of patch complexity. We obtained a consistent spatial distribution of global fire patch functional traits between the MCD64A1 Collection 6 and the MERIS fire_cci v4.1 datasets during their overlap period (2005-2011), confirming the robustness of the applied algorithm and the consistency between both products. This database is relevant to a broad spectrum of fire-related applications such as local to global functional pyrodiversity, fire emissions quantification, and the benchmarking of fire modules embedded in dynamic global vegetation models.

## Background & Summary

Vegetation fires play an important role in the Earth system. They affect vegetation dynamics through biomass burning and post-fire recovery^1^, thus significantly impacting the terrestrial carbon cycle^2^. Gaseous and particulate matter emissions from vegetation fires, including CO_2_, methane, NOx and black carbon^3^, alter atmospheric radiative forcing and in turn the climate. Fire processes and the associated impacts on vegetation are therefore included in Dynamic Global Vegetation Models (DGVM)^4^, which are used to predict future fire activities and vegetation dynamics^5^. Understanding fire processes at a global scale contributes to properly representing biosphere–atmosphere interactions and reliably predicting future climate change and carbon cycle dynamics. Fires, of course, also have strong local economic and health consequences on the human population^6,7^.

Global burned area (BA) products, derived from remote sensing and available since the 2000s, are widely used in various applications given their acknowledged spatial and temporal consistency, and global coverage^8–10^. Remote sensing offers the possibility to map fire-affected areas across the globe, with moderate resolution sensors and frequent revisit time, like the Medium Resolution Imaging Spectrometer (MERIS), or the Moderate-Resolution Imaging Spectroradiometer (MODIS). The reliability of BA retrieval is affected by the quality of input data (through spatial and spectral resolution, and atmospheric correction of the sensor images), the algorithms processing surface reflectance information, and other background observing conditions such as land cover type and cloudiness. Furthermore, the spatial accuracy of BA is critically limited by the spatial resolution of the sensors. Despite these limitations, global satellite data products allow for continuous, frequent and global coverage of burned areas, and are suitable for a systematic analysis of fire dynamics over the globe.

Global fire patch data derived from pixel-level BA information have recently emerged as an important source of information for climate, vegetation and carbon cycle modeling. They are used to capture the key role of driving factors that influence burn area^11^ and benchmark fire module in DGVMs^4^. Process-based fire modules in DGVMs simulate BA as the product of the number of ignitions multiplied by the simulated mean spreading area of a typical fire patch^12–14^, and the results of these models can be compared to global BA products. But this is a less rigorous way to calibrate fire models than a direct comparison between observed and simulated fire patch area, since it is unknown whether a discrepancy between the simulated and observed BA results from differences in the ignition pattern or missing processes in fire propagation. While global or local pyrogeographical analyses could derive significant information from fire patches^15–17^, only a few modeling studies have tried to benchmark fire-DGVM upon fire size distribution^14^ based on local fire patch datasets. In addition to fire patch size distribution, fire patch complexity is another piece of information needed to capture the spreading conditions during fire events. The resulting complexity of fire boundaries also affects post-fire recovery rate in forested area, since it is found that remaining islands of living vegetation^18,19^ can accelerate the recolonization process after a fire. Fire patch size is closely linked to meteorological, fuel continuity and topographical^20^ conditions and can modify carbon emissions according to fire observations in Alaskan boreal forests^21^. Pyrodiversity^22^, which describes the diversity of fire types in a given fire regime, is a promising approach to investigate continental and global spatio-temporal variations in fire spreading processes, recurrence or rarity of events and their drivers. This new field of research in fire science resembles the emerging topic of functional biogeography^23^, where the functioning of biomes is assessed from the assemblage of continuous functional traits rather than species composition and relies on databases of plant traits^24,25^. Similarly, global databases of fire patch morphological features (or traits) that reflect the underlying spreading processes could be used to explore functional pyrogeography as an analytical and modeling framework. Fire regimes could be more precisely described and modeled with an assemblage of fire traits, rather than simple burned area.

This paper presents FRY, a global database of fire patch functional traits that includes patch areas and other morphological features derived from burned areas of the MODIS and MERIS sensors. We first describe the algorithm known as flood-fill^26^ that we use to group individual burned pixels with a burn date into fire patches, defined as groups of adjacent pixels with temporal coincidence parameterized by a temporal cut-off value (section Methods). We extensively compared the functional traits of individual fire patches obtained from the two sensors (section Technical validation), and thoroughly investigated the influence of the cut-off parameter on the measured fire patch functional traits. We concluded that increasing the cut-off value yields larger, longer, and correlatively more complex fire patches without changing the global spatial pattern of the different metrics. Moreover, we showed that the main source of uncertainties on fire patch traits arises from the difference between the two BA products rather than the choice of the cut-off value.

## Methods

### Original burned area input data

The MCD64A1 Collection 6 BA product^27^ is derived from the MODIS sensors onboard the Aqua and Terra satellites. The Terra satellite has a morning overpass time (10h30am), and the Aqua satellite has an afternoon (1h30pm) overpass time. It provides global coverage of burned area with daily revisit at the equator at a resolution of 463x463m over the period 2000-2017. The MCD64A1 Collection 6 algorithm detects BA from daily changes with a burn-sensitive vegetation index computed from the difference between channels 5 and 7 of the spectrometer (corresponding to infrared light) and combined with MODIS active fire observations to discriminate unburned from burned pixels. A burn date, with its uncertainty, is then attributed to each detected burned pixel^27^. The MERIS fire_cci v4.1 BA product is delivered by the European Space Agency Climate Change Initiative Fire Disturbance project (ESA Fire CCI, https://www.esa-fire-cci.org/) using the ENVISAT-MERIS sensor with a resolution of 300x300m and a 10am time of overpass, and covers the period 2005-2011 with a 3-day revisit frequency at the equator. The MERIS fire_cci v4.1 burned area detection algorithm first combines the difference between the measured reflectance in the near-infrared (band 10) and the reflectance in the red (band 8) from the MERIS sensor with active fire observations from MODIS^28^. In a second step, the algorithm uses MERIS reflectance information to grow fire patches from the seed pixels identified during the first step. The final product provides global coverage of burned area every three days (at the equator) from 2005 to 2011 (refs 29,30). Both MCD64A1 Collection 6 and MERIS fire_cci v4.1 datasets are available in separated GeoTIFF tiles, providing the burn date of each burned pixel. Using both datasets allows for comparison of fire patch functional traits calculated from BA. The two burned area products have different advantages, namely a finer spatial resolution for MERIS fire_cci v4.1, and a smaller temporal resolution and longer time series for MCD64A1.

### Construction of fire patches by aggregating neighboring/burned pixels

Here, fire patches were constructed using the ‘flood-fill’ algorithm which aggregates neighboring burned pixels with sequential burn dates into individual fire patches. An important parameter of the algorithm is the cut-off value, defined as the maximum burn date difference between two neighboring pixels considered belonging to the same fire event. The value of this cut-off parameter has an influence on the size of the reconstructed patches, which can differ depending on land cover type and cloudiness at satellite overpass^31^. It is therefore important to test different cut-off values and to analyze their effects on fire patch size distribution and fire morphology. For both MERIS fire_cci v4.1 and MCD64A1 products, fire patches were processed with four different cut-off values of 3, 5, 9 and 14 days. These values were chosen as the possible time lapse between two detected neighboring burned pixels belonging to the same fire event considering uncertainties, so they span the majority of cut-off values used in previous studies^17,26,32^.

The flood-fill algorithm processing daily global burned area data were implemented using the R CRAN package ‘raster’. To optimize the processing time, we made use of the ‘clump’ function which allows pixels of burned areas to be efficiently combined into clumps, defined here as a group of neighboring pixels (in a so-called queen scheme). Working directly at the clump level was more efficient than working at the pixel level and enabled the use of parallelized features of the ‘raster’ package. The algorithm proceeded as follows.

**Figure 1:**
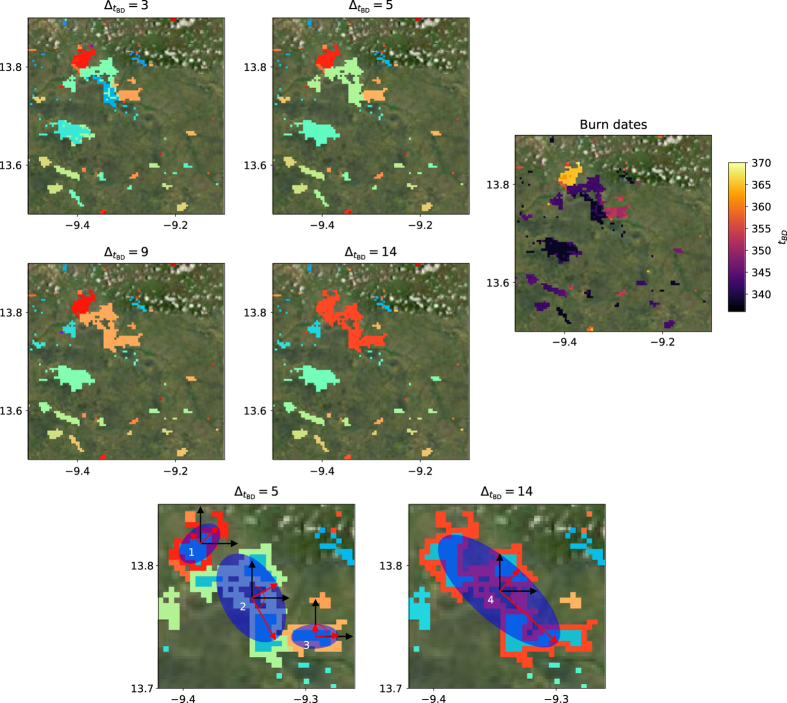
Example of fire patch reconstruction. Figures (**a**) to (**d**) show the fire patches calculated from individual adjacent burned pixels, with temporal coincidence being defined by a temporal cut-off value of 3, 5, 9 and 14, for the MCD64A1 product in a region of northern hemisphere Africa. Continuous areas with the same color indicate the same fire patch. Figure (**e**) shows the burn dates of the pixels (in days). Figures (**f**) and (**g**) show examples of Standard Deviation Ellipses (SDE, dark blue) for three fire patches with cut-off of 5 (zoomed portion of **b**) and one patch with cut-off of 14 (zoomed portion of **d**). The half-axes (σ_X_ and σ_y_) and the azimuthal (θ) angle are displayed in red. The so-called core areas (A_core_, Table 1), corresponding to the area of pixels totally surrounded by other burned pixels in a given patch, are displayed in light blue for the four considered fire patches. Computed values for the functional traits of these patches are shown in Table 2.

A burn date t_bd_ was identified in the BA product, and only the pixels within the time span t_bd_ < t < t_bd_ + Δt_co_ were selected, where Δt_co_ is the cut-off parameter. The clump routine from the R-CRAN raster package was applied to these pixels to attribute a unique ID number to each clump, defined as a group of burned pixels totally isolated by unburned pixels during the considered cut-off dependent time span. We performed the same two previous steps for the next burn date (t_bd_ + 1). When a new clump overlapped with one or more clumps from the previous step, these clumps were considered to belong to the same patch and therefore were merged. For every month, the latitude/longitude coordinates, IDs and burn dates of pixels belonging to a detected fire patch were saved. This information was saved on a monthly time step rather than on a yearly time step since some pixels were susceptible to be detected as burned more than once per year. The resulting fire patches in the same 0.4×0.4º subregion for the four tested cut-off values, with the burn dates associated to the burned pixels, are displayed as an example of the flood-fill algorithm output (Fig. 1).

In order to parallelize the processing of BA into patches, we separated both MCD64A1 and MERIS fire_cci v4.1 products in 3.5×3.5º tiles. To avoid splitting a fire patch that overlaps two tiles, all tile borders overlapped with neighboring tiles with a width of 0.5º. We attributed to a given tile all fire patches whose centers were within the central 2.5×2.5º area, to avoid double counting.

### Calculation of fire patch functional traits

From the monthly patches described above, we used the R CRAN package ‘SDMtools’ (Species Distribution Modeling Tools) to compute all fire patch functional traits including morphological features and spatio-temporal information^32^ (Table 1). The perimeter (P), the total number of burned pixels (N_pixels_) and the number of core burned pixels (N_core pixels_) were computed for each patch, where a core burned pixel is defined as a pixel which is totally surrounded by other burned pixels. These three quantities were computed in pixel units. The corresponding patch areas (in hectares) were also computed (A for N_pixels_, and A_core_ for N_core pixels_). Traits that describe the fire patch complexity are the Perimeter to Area Ratio P.A.R. (=P/N_pixels_), the Shape Index S.I. (=0.25*P/(N_pixels_)^0.5^), the Fractal Correlation Dimension D_2_ (=2*ln(0.25*P)/ln(N_pixels_)) and the Core Area Index C.A. (=A_core_/A). We also provided the minimum and maximum burn date (i.e., corresponding to the date of the first and last detected burned pixel of the fire patch), the mean burn date of the pixels belonging to the same fire patch and the year corresponding to the mean burn date of the fire patch.

The Standard Deviation Ellipse (SDE) was calculated from the spatial coordinates of the burned pixels composing each fire patch, simplifying its geometry to a fitted ellipse by determining the elongation and directional azimuthal angle of the fire patch (Fig. 1). Ellipses are commonly used to simulate theoretical fire patches in most DGVM, for example following the Rothermel equation for fire propagation to compute the ellipse axes^33,34^. The R CRAN package ‘aspace’^35^ was used to compute the center position of the SDE (X and Y), its azimuthal angle (θ), and the minor and major half-axes (σ_X_ and σ_Y_) of the fitted ellipse to each patch (Table 1). The ellipse half-axes and azimuthal angle, initially computed in latitude/longitude coordinates in degrees, were also computed after projecting the fire patch in a local flat projection (σ_X,km_ and σ_Y,km_, in kilometers, and θ_km_). The eccentricity (E_SDE_) and ratio of half-axes of the ellipse (R_SDE_) were also computed. As an example, the values of these different metrics are provided for the patches highlighted in Fig. 1 (Table 2).

The fire patch functional traits were only computed for patches composed of at least 5 burned pixels. This arbitrary threshold allowed us to lighten the database files by removing very small fire patches with unreliable information about the fire patch complexity or orientation.

### Global maps of fire patch functional traits and power law size distribution

Previous studies tried to explain fire patch size distribution with Self-Organized Criticality (SOC)^36,37^. SOC models predict that the distribution of fire patch size follows a power law:

$$N_{f}=\alpha .A_{f}^{-\beta }$$

Where *A*_*f*_ is the area of the fire patch, *N*_*f*_ is the number of fire patches of a given size *A*_*f*_, *α* is a normalization constant and *β* is the exponent of the power law. SOC models usually assume a constant value of *β*, but a number of analyses have detected significant variations of this parameter at a global scale^17,38^. The *β* parameter provides information on asymmetry in the fire patch size distribution. A value *β* = 0 tells us that the number of large fire patches is equal to the number of small fire patches per bin unit; when *β* increases, the frequency of small fire patches increases with respect to large ones. This parameter can be used as an index to investigate potential differences between different burned area products and to test the sensitivity of *β* to different patch reconstruction methods.

We fitted the parameter *β* to the distribution of fire patches in 1º×1º grid cells covering the entire globe during the periods 2005-2011 for MCD64A1 and MERIS fire_cci v4.1. For each cell, we proceeded as follows: first, we produced the profile histogram of N_f_ and A_f_, defined as the normalized number of fires N_f_ for different logarithmic bins of fire patch sizes. We attribute to each value of the profile histogram a Poisson uncertainty equal to the square root of the number of fire patches in each size bin. We then fitted the power law by using the Minuit minimization algorithm (https://seal.web.cern.ch/seal/snapshot/work-packages/mathlibs/minuit/) which provides an efficient way to minimize χ^2^.

As a level 2 product, the maps of *β* and σ_*β*_, the standard deviation of *β* obtained from the fit (computed from the *β* values at χ^2^ ± 1), are also provided because they assess the quality of the fit and allow inter-comparisons between the different datasets. We also produced maps of the mean values and standard deviation for the perimeter-to-area ratio, shape index, fractal correlation dimension, ellipse ratio and ellipse eccentricity, and maps of the number of fire events within each grid cell.

### Code availability

The R-CRAN ‘raster’ (https://CRAN.R-project.org/package=raster), ‘SDMTools’ (https://CRAN.R-project.org/package=SDMTools) and ‘aspace’ (https://CRAN.R-project.org/package=aspace) packages can be found on CRAN, the global repository of open-source packages for R.

Due to the heavy CPU resources required to create the FRY database, the full code has been designed to run on a specific cluster architecture, and providing it would be of limited interest. However, any user interested in creating another database from a pixel BA product can contact the corresponding author who can share snippets of the code and provide help in adapting the code to another architecture.

## Data Records

The FRY database is organized in 8 datasets (2 burned area products for 4 cut-off values), which can be downloaded from the OSU-OREME repository. For each survey SURVEY (MERIS for MERIS fire_cci v4.1 or MODIS_6 for MCD64A1) and each cut-off value N (3, 5, 9 or 14), values for all traits listed in Table 1 are provided for each fire patch in Comma-Separated Format (csv) under the name fire_patches_SURVEY_final_co_N.csv (Data Citation 1). The names and order of the different fields are described in the header of each csv file and in the meta-data associated with the repository.

The NetCDF maps of the parameters described at the end of the Methodology section for the survey SURVEY and the cut-off value N are provided under the name trait_map_SURVEY_1.0deg_co_N.nc (Data Citation 1). The NetCDF files are self-described.

## Technical Validation

**Figure 2:**
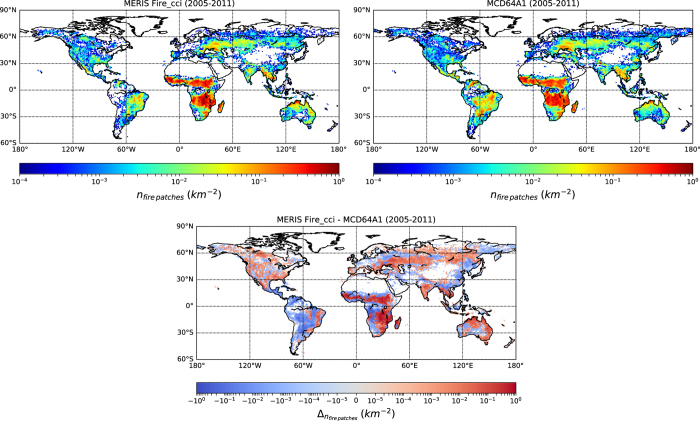
Fire patch density from the fire patch datasets for the years 2005-2011. (**a**) Fire patch density calculated from the MERIS fire_cci v4.1 BA product according to the algorithm described in the Methods section, with a cut-off of 5 days (**b**) Same for the MCD64A1 BA product (**c**) Difference between MERIS fire_cci v4.1 and MCD64A1.

**Figure 3:**
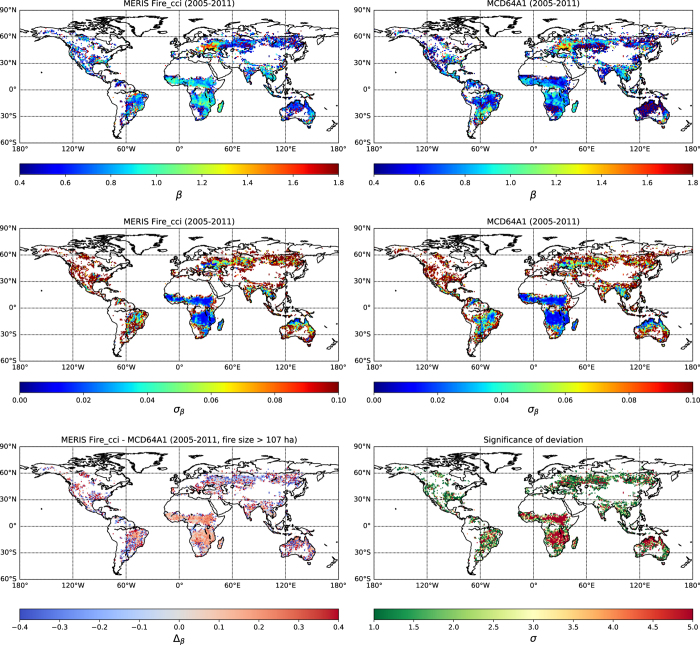
β parameter from the fire patch datasets for the years 2005-2011. (**a**) β parameter calculated from MERIS fire_cci v4.1 BA product with a cut-off of 5 days (**b**) Same for MCD64A1 BA product (**c**) Uncertainty of β for MERIS fire_cci v4.1 for the years 2005-2011 (**d**) Uncertainty of β for MCD64A1 (**e**) Difference between MERIS fire_cci v4.1 and MCD64A1 considering only patches with an area larger than 107 ha (**f**) Level of agreement between the two products (in number of standard errors).

**Figure 4:**
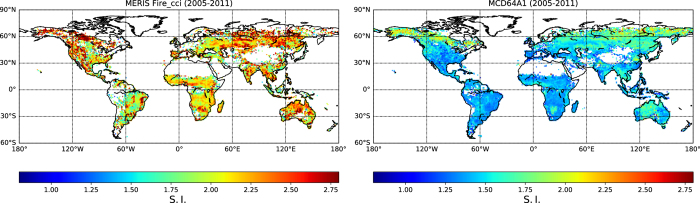
Shape Index (S.I.) from the fire patch datasets for the years 2005-2011. **(a**) S.I. calculated from MERIS fire_cci v4.1 with a cut-off of 5 (**b**) Same for patches calculated with MCD64A1.

In this section, we compare fire patch functional traits between the MERIS fire_cci v4.1 and the MCD64A1 products for 2005-2011. Among the different traits listed in Table 1, we focus on the spatial distribution of the density of individual fire patches (Fig. 2), defined as the number of individual fire patches per square kilometer, the slope of the patch size distribution (*β* parameter, Fig. 3), and the shape index (S.I., Fig. 4) as an indicator of fire patch complexity. We display 1º×1º grid maps of these three metrics for the MCD64A1 and MERIS fire_cci v4.1 with a cut-off value of 5 days. We focused first on the 5 days cut-off value because it corresponds to an intermediate value of the considered cut-off range. We removed from the database all fire patches with less than 5 pixels (Methods section). The actual size of a 5-pixel fire patch in MCD64A1 is larger than a 5-pixel patch in MERIS fire_cci v4.1, and, for a given survey, larger at the equator than at high latitudes. In order to compare fire patches belonging to the same size category, we further removed from MERIS fire_cci v4.1 and MCD64A1 all fire patches smaller than 107 ha, which corresponds to the size of a 5-pixel fire at the equator for a sensor with a 463 x 463m spatial resolution (i.e. the coarser resolution of the two sensors).

### Fire patch density

The distribution of fire patch density is similar between the two products with most of the individual fire patches detected in African tropical savannas, Northern Australia, central Eurasia, Brazilian Cerrado and South-Eastern Asia (Fig. 2). In these regions, MERIS fire_cci v4.1 detects more fire patches than MCD64A1. Conversely, MCD64A1 significantly detects more fires than MERIS fire_cci v4.1 in the other regions of Africa and South America. In total, for a cut-off value of 5, the MERIS fire_cci v4.1 product detected over 2.35 million individual fire events larger than 107 ha, and the MCD64A1 product detected 2.49 million fires larger than 107 ha between the years 2005-2011. When including all detected fire patches (bigger and smaller than 107 ha) for the MERIS fire_cci v4.1 patch data, the fire patch density of the product is larger in all regions. However, the MCD64A1 patch data still indicate more fires in the aforementioned regions. The differences in South America and South-Western Africa most probably arise from the lower number of available MERIS fire_cci v4.1 images in these regions^39^. In boreal regions, the slight discrepancy between the two products is also found when comparing burned area^30^. The lower total number of detected fire patches for MERIS fire_cci v4.1 could arise from missed seeds by the fire_cci algorithm. The 3-day time of overpass can lower the probability of matching burned pixels detected by MERIS imagery with active fire pixels from MODIS if there is a long delay between the two detections. This suggests that the temporal resolution of the sensors is an important parameter for detecting individual fire events when using a two-step algorithm to detect BA.

### Slope of the fire patch size distributions (*β*)

The maps of the fitted *β* parameter for MCD64A1 and MERIS fire_cci v4.1 describing fire patch size distribution were computed with their uncertainties, the difference of *β* between the two products, and their degree of agreement in each cell (Fig. 3). The degree of agreement is measured in units of standard deviation (also noted σ_*β*_) and is equal to N if the fitted values of *β* for a given grid cell are compatible within N σ_*β*_ for the two products. The spatial distribution of *β* is similar between the two fire patch datasets, with higher values in Africa, Eastern Europe (Ukraine and Western Russia) and South Eastern Asia, and lower values in northern Australia, Russia and North America. The uncertainty of the *β* parameter is intrinsically smaller than in regions with a large number of fire events. However, when computing the level of agreement between the products, there are discrepancies in the *β* parameter between the MERIS fire_cci v4.1 and MCD64A1 derived fire patches products in Africa and in North-West Australia, where many cells do not match even within 5 sigmas. These regions correspond to the areas where MERIS fire_cci v4.1 yields more fire patches than MCD64A1, and the power law is usually steeper for MERIS fire_cci v4.1 (higher value of *β*) than for MCD64A1. Under the assumption that the probability of missing big fires is low in both BA products, this means that the MERIS fire_cci v4.1 product detects more small fire patches in these regions with respect to MCD64A1 during the considered time span. It should also be noted that we used Poisson uncertainties for the fit of the power law, which frequently underestimates the uncertainties. This underestimation could degrade the diagnosed level of agreement between the products. Except for these areas, the agreement is fair, with most cells matching within 2 sigmas.

### Shape index (S.I.)

S.I. is systematically higher in the MERIS fire_cci v4.1 product (68% higher on average, Fig. 4). This relative difference does not vary much at global scale and can be explained by the higher spatial resolution of the MERIS sensor, which allows capturing more complex features of fire patches than MCD64A1. This difference can also be linked to the different algorithm used to reconstruct BA: the MERIS fire_cci v4.1 algorithm contains a ‘growing’ phase which was especially designed to reconstruct accurate fire patch shapes. Within each product, S.I. is higher in boreal regions, Australia and South Eastern Asia.

The authors would like to remind potential users of the FRY database that the perimeter of the fire patches are computed from pixel data. All diagonal border lines (NW, NE, SE and SW directions) of the perimeters will appear as ‘stair-step’ lines because of aliasing, and will therefore be biased upward with respect to the real fire patch perimeters. Since this bias depends on the spatial resolution of the pixel product, this renders difficult the direct comparison of the complexity indices between MERIS fire_cci v4.1 and MCD64A1. However, it is still possible to discriminate regions with more complex fire patches from regions with simpler fire patches for products with similar resolution.

### Sensitivity to varying the cut-off parameter on regional statistics of fire patch functional traits

**Figure 5:**
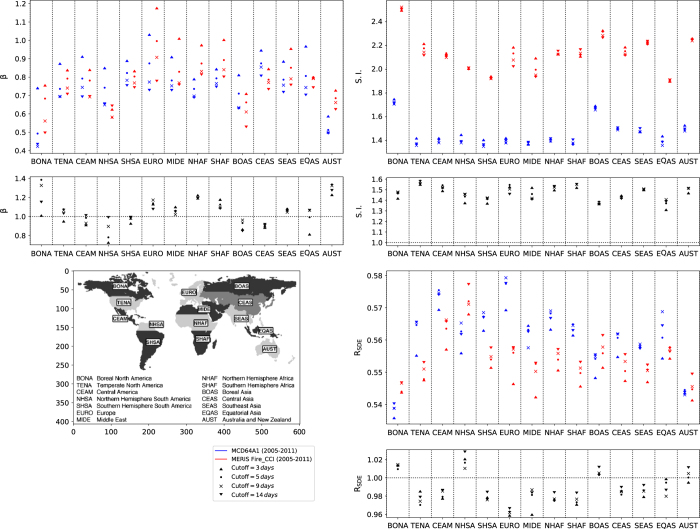
Median values of *β* parameter, shape index and ellipse ratio for different GFED regions. The values are displayed for MERIS fire_cci v4.1 and MCD64A1, and for the 4 different cut-off values. The bottom plot displays the ratio of the median GFED values between the two products (MERIS fire_cci v4.1 divided by MCD64A1) for each displayed fire patch functional traits.

We next compare the values of *β*, S.I. and R_SDE_ averaged over the 14 Global Fire Emissions Database (GFED) regions for the 4 considered cut-off values (Fig. 5). To compute the average, the mean values in the 1º×1º cells are weighted by their uncertainties. We also display the ratio of the average values obtained for MERIS fire_cci v4.1 and MCD64A1. The cut-off value is arbitrarily chosen to reconstruct the fire patches. Here, we use the dispersion of the different cut-off values as a rough estimation of the uncertainty due to the arbitrary choice of the cut-off parameter value to reconstruct fire patches.

The value of the *β* parameter decreased as the cut-off value increased. Increasing the cut-off tends to group together fire patches which would have been separated with a lower cut-off. As a result, the number of small fires decreases and the number of large fires increases for increasing cut-off values (Fig. 1), resulting in a steeper power law for small cut-off values. If we consider the dispersion of *β* for the 4 tested cut-off values, *β* values are in agreement between the two products for a given cut-off value, except for Northern Hemisphere Africa (NHAF) and Australia (AUST). As mentioned above, the difference between the two products is emphasized by the underestimation of the uncertainty when performing the fit of the *β* parameter.

**Figure 6:**
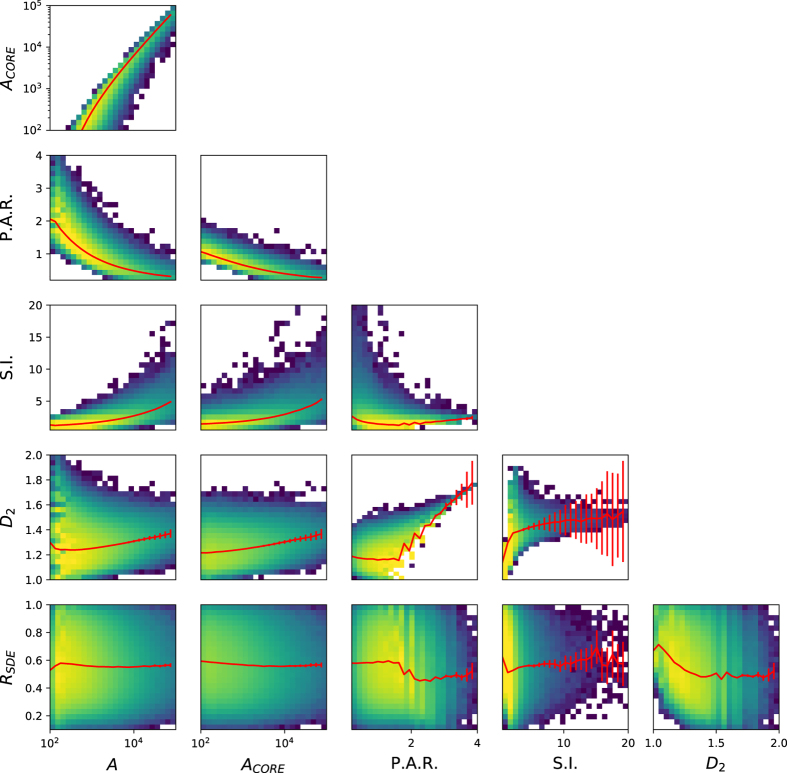
Profile histograms between different fire patch functional traits for a cut-off value of 5. The red lines represent the weighted mean of the ordinate in different bins of the abscissa, and the error bars the standard deviation on the weighted mean. The error bars are sometimes not noticeable because they are smaller than the width of the line.

Increasing the cut-off value produces patches with a higher shape index, which corresponds to more complex fire patches. This effect is probably linked to the significant correlation between fire patch size and shape index (ρ = 0.405, p-val < 0.001, Fig. 6). Larger and longer fires can experience more variations in wind orientation while they burn, and are therefore susceptible to have a shape much different from a simple ellipse. When comparing the average values of S.I. across the GFED regions, S.I. is higher in boreal regions for both products. This effect probably arises from the gridded BA pixel product: a fire patch of a given size will be composed of more pixels in high latitudes than at the equator, which will result in an artificially more complex shape.

Higher cut-off values also yielded higher R_SDE_. As for the correlation between S.I. and patch size, we would expect longer and larger fires to propagate further and have more elongated ellipses than small fire patches. The correlation between A and R_SDE_ was found to be close to zero, but there is a slight correlation between R_SDE_ and core area (Fig. 6). This can be explained by the fact that the SDE is defined to contain 66% of the burned pixels of a fire patch. A core area pixel has a higher probability to be located within the ellipse. R_SDE_ is therefore more sensible to the core area than the total area of the fire patch. MCD64A1 fire patches are slightly more elongated (~1-2%) than MERIS fire_cci v4.1, except for boreal regions and NHSA, whereas the MERIS fire_cci v4.1 product yields slightly higher ellipse ratio than MCD64A1.

## Additional information

**How to cite this article**: Laurent, P. *et al*. FRY, a global database of fire patch functional traits derived from space-borne burned area products. *Sci. Data* 5:180132 doi: 10.1038/sdata.2018.132 (2018).

**Publisher’s note**: Springer Nature remains neutral with regard to jurisdictional claims in published maps and institutional affiliations.

## Supplementary Material



## Figures and Tables

**Table 1:** List of the output fire patch functional traits of the FRY database.

Patch ID	ID number of the fire patch
N_pixels_	Total number of burned pixels
N_core pixels_	Number of core burned pixels
A	Area of the fire patch (ha)
A_core_	Core area of the fire patch (ha)
P	Perimeter of the fire patch (pixel sides)
P.A.R.	Perimeter to Area Ratio (= P/N_pixels_)
S.I.	Shape Index (= 0.25*P/(N_pixels_)^0.5^)
D_2_	Fractal Correlation Dimension ( =2*ln(0.25*P)/ln(N_pixels_))
C.A.	Core Area index (= A_core_/A)
X	Longitudinal coordinate of the fire patch center (deg)
Y	Latitudinal coordinate of the fire patch center (deg)
σ_X_	Small half axis of the SDE (deg)
σ_y_	Big half axis of the SDE (deg)
θ	Rotation angle of the ellipse (in lat/long coordinates). Corresponds to the angle between north and σ_Y,_ (deg)
σ_X,km_	Small half axis of the SDE (km)
σ_Y,km_	Big half axis of the SDE (km)
θ_km_	Rotation angle of the ellipse (in flat projection). Corresponds to the angle between north and σ_Y,km_ (deg)
R_SDE_	Ellipse Ratio ( = σ_X,km_/σ_Y,km_,0 < R_SDE_ < 1)
E_SDE_	Ellipse Eccentricity (= (1 – min(σ_X,km_,σ_Y,km_)^2^/max(σ_X,km_,σ_Y,km_)^2^))^0.5^)
BD_min_	Minimum pixel burn date of the fire patch
BD_max_	Maximum pixel burn date of the fire patch
BD_mean_	Mean value of pixel burn date of the fire patch
Year	Year associated with Mean_BD

**Table 2:** Values of the different functional traits for the four patches shown in Fig. 1f,g.

Patch	Fire patch 1	Fire patch 2	Fire patch 3	Fire patch 4
N_pixels_	63	171	49	295
N_core pixels_	21	51	16	99
A (ha)	146.40	397,44	113.90	685.6
A_core_ (ha)	48.80	118.53	37.19	230.1
P	52	136	42	220
P.A.R.	0.825	0.795	0.857	0.746
S.I.	1.625	2.51	1.5	3.14
D_2_	1.24	1.37	1.21	1.41
C.A.	0.33	0.30	0.33	0.40
X (deg)	−9.39	−9.34	−9.29	−9.34
Y (deg)	13.82	13.77	13.74	13.78
σ_X_ (deg)	0.012	0.024	0.009	0.024
σ_Y_ (deg)	0.020	0.039	0.019	0.063
σ_X,km_ (km)	1.31	2.57	1.03	2.60
σ_Y,km_ (km)	2.15	4.30	2.07	6.90
R_SDE_	0.61	0.60	0.51	0.38
E_SDE_	0.79	0.80	0.85	0.93
θ (deg)	46.5	150.6	87.1	131.9
The selected fire patches are annotated by their corresponding numbers in Fig. 1f,g.				
